# Use of clinic- and community-based overdose prevention services by sex workers who use drugs: findings from a community-based cohort in Vancouver, Canada (2017–2024)

**DOI:** 10.1186/s12954-026-01398-x

**Published:** 2026-01-27

**Authors:** Sarah Moreheart, Kate Shannon, Kanna Hayashi, Wiebke Bartels, Jennie Pearson, Andrea Krüsi, Shira Miriam Goldenberg

**Affiliations:** 1 Faculty of Health Sciences, Simon Fraser University, 8888 University Drive, Burnaby, BC V5A 1S6 Canada; 2 Department of Medicine, University of British Columbia, 2755 Laurel Street, Vancouver, BC V5Z 1M9 Canada; 3 School of Population and Public Health, University of British Columbia, 2206 East Mall St, Vancouver, BC V6T 1Z3 Canada; 4 Department of Criminology, Faculty of Arts, Simon Fraser University, 8888 University Drive, Burnaby, BC V5A 1S6 Canada; 5 Division of Epidemiology and Biostatistics, School of Public Health, San Diego State University, 5500 Campanile Drive, San Diego, CA 92182-4162 USA; 6 BC Centre on Substance Use, 400-1045 Howe Street, Vancouver, BC V6Z 2A9 Canada; 7 Department of Division of Infectious Diseases and Global Public Health, Department of Medicine, University of California, 9500 Gilman Dr. La Jolla, San Diego, CA 92093 USA

**Keywords:** Sex workers, Drug toxicity crisis, Naloxone, Overdose prevention

## Abstract

**Background:**

Sex workers who use drugs are disproportionately impacted by the current overdose crisis and face many structural barriers to health and harm reduction services. Delivered in either community (e.g., embedded in supportive housing) or clinic (e.g., hospitals) settings, overdose prevention services are crucial harm reduction interventions. Gaining insight into which services sex workers use, and whether sex worker-specific programs impact this use, is key to identifying targeted prevention strategies and enhancing the continuum of overdose care for this population.

**Methods:**

Data were derived from An Evaluation of Sex workers’ Health Access (AESHA), a prospective, community-based cohort of women (trans-inclusive) sex workers in Vancouver, Canada (March 2017–March 2024). We plotted biannual trends in use of overdose prevention services, comparing community-based and clinic-based services (Aim 1), and used generalized linear mixed models to characterize uptake of overdose prevention services, including potential differences between community and clinic-based services (Aim 2). Finally, we evaluated the association between use of sex worker-specific programs and uptake of overdose prevention services, including potential differences between community or clinic-based services over the 7-year study period (Aim 3).

**Results:**

Among 503 sex workers who used drugs, 82.1% (*N* = 413) used any overdose prevention services over the seven-year study. Uptake of community-based versus clinic-based overdose prevention services was 70.2% (*N* = 353) and 60.2% (*N* = 303), respectively. Use of sex worker-specific programs was positively associated with use of overdose prevention services (adjusted odds ratio [AOR] 2.18, 95% confidence interval [CI] 1.82–2.62), and this association was strongest for community-based services (AOR 2.66, 95% CI 2.21–3.20) as opposed to clinic-based services (AOR 1.73, 95%CI 1.41–2.13).

**Conclusion:**

Uptake of overdose prevention services among sex workers is relatively high, but faced concerning declines over the study period, highlighting the need for additional interventions to scale-up access. Use of sex worker-specific programs facilitated wider access to overdose prevention services. Findings underscore the importance of expanding sex worker-specific and peer-led programs as part of scaling up overdose prevention efforts.

**Supplementary Information:**

The online version contains supplementary material available at 10.1186/s12954-026-01398-x.

## Highlights

Sex worker-programs facilitated use of overdose prevention services.

Higher cumulative prevalence observed for community-based services.

Use of overdose prevention services have not returned to pre-pandemic levels.

**Supplementary Information:**

The online version contains supplementary material available at 10.1186/s12954-026-01398-x.

## Introduction

The surge in overdose events and fatalities in North America since 2016, driven by the toxicity of substances sourced from unregulated drug markets, has prompted the need for public health responses [1, 2]. British Columbia (BC) has been particularly affected, leading to the declaration of a provincial public health emergency in April 2016 [3]. As part of harm reduction practices, overdose prevention services are interventions designed to reduce the risk of drug overdose, improve response to overdose incidents, and prevent fatalities. These services are pragmatic, voluntary, non-punitive approaches which aim to support individuals who use drugs by providing education, resources, and tools to reduce overdose risks and promote safer drug use practices [4]. Overdose prevention services include offerings and resources such as monitored drug consumption, drug checking services (where one can submit a drug sample and receive an analysis of the drug composition), naloxone distribution (i.e., take-home naloxone [THN] kits and training), information and support on safer drug-taking practices (e.g., instructions on how to inject), as well as information and referrals to health or social services, which may be delivered or rendered by clinical staff (e.g., a nurse), community-support staff (e.g., a supportive housing worker), or a fellow person who uses drugs (PWUD) [5, 6].

Overdose prevention services can be categorized as either *community-based* or *clinic-based*. Community-based overdose prevention services are non-clinical, low-barrier locations (e.g., housing, drug-using sites) that operate outside formal healthcare settings. These services are driven by unmet needs in clinical care, such as the demand for overdose prevention support for inhalation. They are delivered by non-medical/allied staff (e.g., frontline workers) and people who use drugs (i.e., “peers”) [7–9]. Clinic-based overdose prevention services are operated by medical/allied-healthcare providers (e.g., nurses, social workers) that take place in clinical locations (e.g., hospitals and pharmacies). These services offer a structured approach that may include additional healthcare resources, such as wound care, mental health support, or linkage to treatment. While some clinic-based programs incorporate peers in service delivery, they operate within a regulated healthcare framework, distinguishing them from community-based models [10]. Systematic reviews have consistently shown that overdose prevention services are effective in achieving their goals, including reducing harms associated with drug use such as preventing blood-borne diseases, lowering overdose risks and related harms, improving the health of people who use drugs, and increasing referrals to health and social services [11–14]. During the COVID-19 pandemic, many overdose prevention services in Vancouver experienced temporary closures, reduced hours, or modified operations, which disrupted access [15, 16]. Overdose prevention services provided by community-based programs have demonstrated greater reach compared to services provided by formal institutions (e.g., public health departments) [17]. Further, peer-inclusive services are associated with creating trusted overdose prevention services and are integral to the acceptability of such services among people who use drugs [8, 18, 19]. However, little documented research has examined access to and outcomes of overdose prevention measures among sex workers, including identifying individual and structural factors associated with access to community and clinic-based services.

Sex workers have been disproportionately affected by the overdose crisis. Longitudinal studies in Vancouver, BC report a cumulative prevalence of 27.6% for nonfatal overdoses among sex workers over 7.5 years [20], as well as a rising incidence of first nonfatal overdoses over a 9-year period (2010–2019), with criminalization-related barriers identified as key predictors [21]. Additionally, research shows that among people who inject drugs, sex workers face 2.21 times higher odds of experiencing a nonfatal overdose compared to non-sex workers [22]. Amidst pervasive structural marginalization, sex workers are vulnerable to overdose morbidity and mortality and other adverse outcomes such as gender-based violence [23] and limited access to health and social services [20, 24, 25]. Sex workers who use drugs face the burdens of criminalization due to their involvement in both sex work and criminalized drug use, and are burdened with health and social disparities [26–28]. The intersecting challenges encountered by sex workers underscore the necessity for tailored, gender-specific interventions [29–31], particularly considering the significant proportion of sex workers globally who engage in drug use [32]. Sex worker-led interventions are one such approach and have repeatedly demonstrated positive outcomes to optimize sex workers’ health, safety, and human rights, and are recognized and endorsed as a best practice to address the health and safety of sex workers [33–37]. In BC, sex worker-led and/or sex worker-specific programs offer services such as drop-in centres, distribution of harm reduction and safer sex supplies, advocacy and more in a safe, confidential, non-judgmental space [38], and are typically operated by and for sex workers. Despite the scientific evidence supporting the effectiveness of such interventions to impart occupational health, legal, and safety supports (e.g., career and legal counselling, safety planning and bad date lists) [39–43], the impact of the use of sex worker-specific programming on use of overdose prevention services has not yet been examined.

Given the substantial impact of the overdose crisis on highly marginalized sex workers [21, 22], effective service plan design and delivery requires longitudinal data to determine the trends and characterize patterns of uptake of overdose prevention services by sex workers. Previous work examining overdose prevention service use and characteristics of sex workers who access these services is limited and to date has been mainly qualitative [44–47]. Little is known about the long-term overdose prevention service use patterns among sex workers who use drugs, particularly regarding the factors associated with accessing different types of services (e.g., community-based, clinic-based). Addressing this gap is essential to improving accessibility, adapting to evolving needs, and informing evidence-based policies and resource allocation to mitigate overdose-related harms. Although earlier work has documented the essential role of peer-led programming for effective and acceptable services for people who use drugs [19, 48, 49], previous studies have not examined whether programming that is sex worker-focused influences engagement with overdose prevention services for this population. This study seeks to fill these gaps through three aims: (1) to describe period prevalence and time trends in use of overdose prevention services, including community- and clinic-based services; (2) to longitudinally characterize uptake of overdose prevention services, including identifying individual and structural factors associated with access to community and clinic-based services; and (3), to longitudinally evaluate the association of use of sex worker-specific programs and access to any overdose prevention services, any community-based services, and clinic-based services over seven-years (2017–2024).

## Methods

Data were derived from An Evaluation of Sex Workers Health Access (AESHA), a prospective, open cohort including 900 + sex workers who work across diverse environments in Metro Vancouver (e.g., indoor, street-based, and online-advertising sex workers), which recruited participants from 2010–2024. This study was developed on the basis of collaborations with local sex work agencies since 2005 [50] and continues to be monitored by a Community Advisory Board of representatives of 15 + community agencies. Eligibility criteria at enrolment included identifying as a cisgender or transgender woman^1^ over 14 years of age, exchanging sex for money within the last 30 days, and being able to provide written informed consent. The majority of participants in the AESHA cohort were engaged in in-person sexual services, primarily within indoor venues such massage parlours and outdoor/street-based settings, reflecting the main contexts in which sex work occurs within this cohort, as documented in prior publications [51–53]. Time-location sampling was used to recruit youth and adult cisgender and transgender women engaged in recent sex work through day and late-night outreach to outdoor/public sex work locations (i.e., streets, alleys) and indoor sex work venues (i.e., massage parlours, micro-brothels, and in-call locations) across Metro Vancouver [50]. In addition, online recruitment was used to reach sex workers in online solicitation spaces. Indoor sex work venues and outdoor solicitation spaces (‘strolls’) were identified through community mapping conducted together with current/former sex workers and updated by the outreach team on an ongoing basis as needed. The study holds ethical approval through the Providence Health Care/University of British Columbia Research Ethics Board.

### Data collection

As questions on overdose prevention access were added in 2017, analyses were restricted to participants who used drugs (self-reported use of illicit or non-prescribed substances [e.g., opioids, stimulants, or sedatives] by any route of administration) and completed semi-annual questionnaire data collected between March 2017 and March 2024. At each visit, study questionnaires were administered in the community-based research office or a confidential location of participants’ choice (e.g., within their home or at a sex work location) by trained interviewers/outreach workers with lived (i.e., current/former sex work) and/or community experience (i.e., professional or volunteer experience working directly with the population involved in the study—such as providing outreach, support, advocacy, or services within that community) via REDCap [54, 55]. During the initial phase of the COVID-19 public health emergency, some study visits were temporarily conducted remotely (via phone), based on institutional and public health guidance for safe research during the first year of the pandemic. The main questionnaire included questions on individual socio-demographic characteristics (e.g., age, minoritized gender identity [e.g., cisgender vs transgender woman identity, inclusive of transfeminine identities^1^]); minoritized sexual identities (e.g., 2SLGBQ+ vs heterosexual), drug use, sex work patterns, and structural factors including violence, the work environment, criminalization and policing, sex work community mobilization, and access to health, substance use, social, and other services. Additionally, study visits included voluntary sexual health visits including HIV/STI/HCV serology testing by a sexual health nurse at the study office or at a safe location identified by participants, along with referral/treatment as needed. At inception (January 2010), participants received a $40 honorarium for each baseline or semi-annual follow-up visit. From September 2021 to March 2022, this amount was adjusted to $65 per visit, given the increased burden to participants of revised questionnaires which were expanded to address impacts of the COVID-19 pandemic. In May 2022, this was further increased to $80 per baseline visit and $65 per semi-annual follow up visit, to account for inflation and participant burden related to questionnaire length. Participants are offered connections to relevant health and social services at each visit as needed, alongside harm reduction supplies and other necessities.

### Study variables

Use of overdose prevention services (e.g., drug checking services, monitored drug consumption, etc.) was defined as a binary (yes vs. no) to the question “have you accessed any of the following sites for overdose prevention services?” Response options included local overdose prevention services designed to reduce the risk of drug-related overdoses through harm reduction strategies, education, THN distribution, supervised consumption, and connections to treatment and support services well as free-text responses. Because the study questionnaire asked broadly about ‘overdose prevention services’ without disaggregating by type, responses did not distinguish whether participants accessed supervised drug consumption or drug checking. In addition, stand-alone drug checking services were not reported in our dataset, while some overdose prevention sites co-located both types of services. For these reasons, supervised drug consumption and drug checking were analyzed as a combined outcome variable.

All responses were then categorized into either *community* or *clinic*-based services. *Community-based* services are defined as non-clinical low-barrier locations (e.g., housing, drug-user run service organizations that provided peer monitoring of drug consumption) that are operated and delivered by non-medical/allied staff (e.g., frontline workers) and peers. *Clinic-based* services are defined as service provision delivered by medical/allied-healthcare providers (e.g., nurses, social workers) that take place in clinical locations (e.g., hospitals and pharmacies) and may or may not employ peers in the delivery of their services (Supplementary Table 1). To plot biannual trends in use of overdose prevention services, comparing community-based and clinic-based services (Aim 1), three separate outcome variables were developed assessing access to: 1) any overdose prevention services (i.e., either any community-based or clinic-based service vs. no use of services); 2) community-based overdose prevention services only (yes vs. no, where the ‘no’ category includes those who used only clinic-based services or accessed no services); and 3) clinic-based overdose prevention services only (yes vs. no, where the ‘no’ category includes those who used only community-based services or accessed no services).

To characterize uptake of overdose prevention services and identify individual and structural factors associated with accessing community or clinic-based services (Aim 2), independent variables were determined a priori based on a review of the literature with sex workers and women who use drugs. These variables included individual factors as well as structural determinants hypothesized to be associated with engagement with overdose prevention services. Apart from time-fixed demographic characteristics including age and racialization, all other variables used in this study were evaluated as time-varying with occurrences updated at each semi-annual follow-up which examined events during the past six months or current measures at the time of the study visit. *Individual characteristics* included age and racialization, which was coded as Indigenous identity (includes First Nations, Metis, and Inuit), Woman of Colour (e.g., Black, Asian, Latina), and White.^2^ Time-updated demographics included identifying as a minoritized gender identity (e.g., transgender vs. cisgender), and identifying as a minoritized sexual identity (LGBTQ2S vs heterosexual). Drug use patterns included having needed help with injecting (yes vs. no vs. N/A-no injection drug use), route of consumption (e.g., injection or non-injection), and experiences of non-fatal overdose over one’s lifetime and in the last six months.

*Structural determinants* included time-updated questions measuring living environments. Precarious housing was defined as insecure, overcrowded, or substandard accommodations without legal tenure or stable rental agreements [56]. In this study, this was operationalized as a binary variable (yes vs. no) and included living in a single room occupancy hotel (SRO),^3^ staying with family/relatives, supportive housing, modular housing or homelessness. Work environment measures included several variables. First, the primary place of solicitation, categorized as independent (e.g., own or client’s home, hotels/motels, other informal spaces outside of managed venues), formal indoor (e.g., massage parlour), or street/public. Second, the primary place of providing services for sex work, categorized as outdoor/public, informal indoor (e.g., drug house, nightclub, in-call at own apartment, out-call at client’s place), formal indoor (e.g., massage parlour, brothel, sex worker-supportive housing). Finally, drug use in the context of sex work, which was defined as answering yes to a question about using drugs with a sex work client during a sex work transaction (yes vs. no). To characterize interactions with the criminal legal system, variables were included which captured negative police encounters while doing sex work and whether a participant experienced police-related barriers to harm reduction (e.g., difficulty accessing drugs, clean rigs, other drug equipment, rushing drug use, confiscation of drug equipment, money or drugs, ‘jacked up by cops’). Other structural variables included barriers to healthcare, enrollment in opioid agonist therapy (e.g., methadone, injectable buprenorphine, buprenorphine-naloxone, injectable hydromorphone, injectable diacetylmorphine, other prescription opioids for off-label addiction treatment) and having accessed any other alcohol/drug treatment (yes vs. no).

For Aim 3, the explanatory variable of interest was *use of sex worker-specific programs*, which was coded as a binary variable (yes vs. no) in response to the question, “have you ever used any of the following health and support services?” Sex worker-specific or led programs were defined as drop-in centres and mobile outreach services operated by and for sex workers that provide low-threshold supports in a safe, confidential, and non-judgmental environment. These offered low-threshold services, such as hot meals, showers, hygiene items, clothing, harm reduction and safety supplies (e.g. bad date sheets, condoms), and referrals to social and health support services. Responses indicating use of these sex worker-specific or sex worker-led programs were coded as ‘yes.’ Participants who did not use any of these were coded as ‘no.” Outcome variables included use of overdose prevention services (either any community and/or any clinic-based service), using any community-based services only, or using any clinic-based services only, as defined in Aim 1. Confounder selection was based on the literature and included structural and individual factors known to be associated with the exposure and outcomes.

### Statistical analyses

Analyses were restricted to participants who reported having ever used injection or non-injection drugs (excluding cannabis and alcohol) and participated in the study between March 1, 2017 and March 31, 2024. Drug use was defined as self-reported use of illicit or non-prescribed substances such as heroin, fentanyl, methamphetamine, cocaine, crack cocaine, non-prescribed opioids (e.g., hydromorphone), benzodiazepines, MDMA, ketamine, and GHB, via injection or non-injection routes (e.g., oral, nasal, smoked, or injected). Unadjusted odds ratios (OR) and adjusted odds ratios (AOR) with 95% confidence intervals (CI) are reported. All analyses were performed in SAS version 9.4 (SAS, Cary, NC, USA) and R version 4.4.1.

#### Use of overdose prevention services over time (aim 1)

Point prevalence was calculated at six-month follow-up intervals as the proportion of participants reporting use of overdose prevention services across the three outcomes: use of any overdose prevention services (*either* any community-based or any clinic-based); use of any community-based overdose prevention services; and use of any clinic-based prevention services. Trends over time were examined using ordinary least squares (OLS) regression of semiannual proportions across follow-up intervals. The Durbin–Watson test and inspection of autocorrelation and partial autocorrelation plots were used to assess serial correlation in model residuals. As no substantial autocorrelation was detected, autoregressive correction was not required. Slope estimates and 95% confidence intervals (CIs) were reported to evaluate changes over time in each outcome.

#### Characterize uptake of overdose prevention services (aim 2)

Explanatory individual and structural variables of interest were stratified by our three outcomes, as outlined above. Descriptive analyses drew on participants’ first available observation and compared those who accessed overdose prevention services and those who did not, using Pearson's chi-squared test for categorical variables (or in the case of small cell counts, Fisher's exact test) and the Wilcoxon rank-sum test for continuous variables. Bivariate analysis used generalized linear mixed-effects models (GLMM), using a logit link function to reflect the binary nature of the outcome. To account for differences between individuals and the correlation within repeated measurements, we included random intercepts in the models, capturing individual-level variability. We adjusted the subject-specific estimates to reflect population-level odds ratios using the marginal_coefs function from the GLMMadaptive R package [60]. At the bivariate analysis stage the variable ‘primary place of sex work service’ was collapsed from four to three levels through combining the *informal indoor* and *formal indoor* categories into one (*indoor (formal/informal)* due to the low cell counts of the *formal indoor* category.

#### Use of sex worker-specific programs and use of overdose prevention services (aim 3)

Multivariable analysis using GLMM was employed to longitudinally evaluate the association between the use of sex worker-specific programs and use of overdose prevention services. Listwise deletion was used to handle missing data. Separate multivariable models were developed for each of the three outcomes. Each model adjusted for the same set of covariates selected a priori; variables that had a high degree of collinearity were excluded.

## Results

The study sample included 503 sex workers who used drugs (self-reported use of illicit or non-prescribed substances [e.g., opioids, stimulants, or sedatives] by any route of administration) who completed a median number of 5 visits (IQR 2–9) and contributed 2955 observations between 2017 and 2024. Among these, 55.7% (*N* = 280) used any overdose prevention service, 35.6% (*N* = 179) used any community-based service, and 35.4% (*N* = 178) used any clinic-based service at the time of their first available observation. At the participant’s first available observation (Table 1), the median age was 40 years old (interquartile range [IQR] = 33–48), and 54.5% (*N* = 274) reported identifying as a minoritized sexual identity. More than half (55.7%; *N* = 280) identified as Indigenous. Most participants (86.9%; *N* = 437) reported being precariously housed in the last six months and 23.5% (*N* = 118) reported being homeless in the last six months. Over half (57.5%, *N* = 289) reported a previous non-fatal overdose. Nearly one fifth (19.9%, *N* = 100) reported needing injection assistance in the last six months.

**Table 1:** Participant characteristics stratified by uses of any overdose prevention services, any community-based overdose prevention services, and any clinic-based overdose prevention services in the last 6 months among sex workers who use drugs in Metro Vancouver, Canada (*N* = 503), AESHA 2017–2024

			Used any overdose prevention services (community or clinic)*		Used any community-based overdose prevention services*		Used any clinic-based overdose prevention services*	
Characteristic	Total (%) (*N* = 503)	Missing (%)	Yes (%) (*N* = 280)	No (%) (*N* = 223)	Yes (%) (*N* = 179)	No (%) (*N* = 324)	Yes (%) (*N* = 178)	No (%) (*N* = 325)
Structural determinants								
Precariously housed*	437 (86.9)	1 (0.2)	255 (91.1)	182 (81.6)	164 (91.6)	273 (84.3)	164 (92.1)	273 (84.0)
Homeless*	118 (23.5)		71 (25.4)	47 (21.1)	50 (27.9)	68 (21.0)	47 (26.4)	71 (21.9)
Primary place of solicitation*		13 (2.6)						
Outdoor/public space	163 (32.4)		116 (41.4)	47 (21.1)	75 (41.9)	88 (27.2)	74 (41.6)	89 (27.4)
Indoor (informal or formal)	34 (6.8)		14 (5.0)	20 (9.0)	11 (6.2)	23 (7.1)	7 (3.9)	27 (8.3)
Independent	144 (28.6)		73 (26.1)	71 (31.8)	44 (24.6)	100 (30.9)	52 (29.2)	92 (28.3)
N/A-didn’t solicit in the last 6 months	149 (29.6)		71 (25.4)	78 (35.0)	44 (24.6)	105 (32.4)	42 (23.6)	107 (32.9)
Primary place of sex work service*		10 (2.0)						
Outdoor/public space	149 (29.6)		101 (36.1)	48 (21.5)	71 (39.7)	78 (24.1)	66 (37.1)	83 (25.5)
Informal indoor	180 (35.8)		101 (36.1)	79 (35.4)	60 (33.5)	120 (37.0)	67 (37.6)	113 (34.8)
Formal indoor	15 (3.0)		1 (0.4)	14 (6.3)	1 (0.6)	14 (4.3)	0 (0.0)	15 (4.6)
N/A-didn’t solicit in the last 6 months	149 (29.6)		71 (25.4)	78 (35.0)	44 (24.6)	105 (32.4)	42 (23.6)	107 (32.9)
Used any drugs with client during a sex work transaction*	180 (35.8)	28 (5.6)	112 (40.0)	68 (30.5)	76 (42.5)	104 (32.1)	76 (42.7)	104 (32.0)
Used sex worker-specific program*	275 (54.7)	1 (0.2)	187 (66.8)	88 (39.5)	129 (72.1)	146 (45.1)	121 (68.0)	154 (47.4)
Worked with other sex workers for safety*		3 (0.6)						
Yes	54 (10.7)		34 (12.1)	20 (9.0)	22 (12.3)	32 (9.9)	23 (12.9)	31 (9.5)
No	297 (59.1)		173 (61.8)	124 (55.6)	111 (62.0)	186 (57.4)	112 (62.9)	185 (56.9)
N/A-no sex work in last 6 months	149 (29.6)		71 (25.4)	78 (35.0)	44 (24.6)	105 (32.4)	42 (23.6)	107 (32.9)
Experienced violence from community members/businesses*	41 (8.2)	10 (2.0)	32 (11.4)	9 (4.0)	24 (13.4)	17 (5.3)	19 (10.7)	22 (6.8)
Experienced physical/sexual violence from a sex work client*	36 (7.2)	19 (3.8)	27 (9.6)	9 (4.0)	17 (9.5)	19 (5.9)	17 (9.6)	19 (5.9)
Experienced violence from negative police encounters*	75 (14.9)	5 (1.0)	54 (19.3)	21 (9.4)	34 (19.0)	41 (12.7)	39 (21.9)	36 (11.1)
Experienced any barriers to harm reduction due to police presence*	83 (16.5)	15 (3.0)	59 (21.1)	24 (10.8)	42 (23.5)	41 (12.7)	42 (23.6)	41 (12.6)
Experienced any healthcare barriers*	302 (60.0)	6 (1.2)	173 (61.8)	129 (57.9)	117 (65.4)	185 (57.1)	115 (64.6)	187 (57.5)
Enrollment in opioid agonist therapy*		15 (3.0)						
Yes	218 (43.4)		130 (46.4)	88 (39.5)	86 (48.0)	132 (40.7)	83 (46.6)	135 (41.5)
No	207 (41.2)		122 (43.6)	85 (38.1)	74 (41.3)	133 (41.1)	82 (46.1)	125 (38.5)
N/A-never used opiates	63 (12.5)		19 (6.8)	44 (19.7)	13 (7.3)	50 (15.4)	8 (4.5)	55 (16.9)
Accessed drug/alcohol treatment*	197 (39.2)	8 (1.6)	128 (45.7)	69 (31.0)	81 (45.3)	116 (35.8)	85 (47.8)	112 (34.5)
Overdosed*	68 (13.5)	7 (1.4)	53 (18.9)	15 (6.7)	34 (19.0)	34 (10.5)	42 (23.6)	26 (8.0)
Lifetime overdose experience	289 (57.5)	9 (1.8)	185 (66.1)	104 (46.6)	119 (66.5)	170 (52.5)	119 (66.9)	170 (52.3)
Non-injection drug use†*	337 (67.0)	6 (1.2)	203 (72.5)	134 (60.1)	131 (73.2)	206 (63.6)	128 (71.9)	209 (64.3)
Injection drug use*	266 (52.9)	3 (0.6)	192 (68.6)	74 (33.2)	118 (65.9)	148 (45.7)	142 (79.8)	124 (38.2)
Needed injection assistance*	100 (19.9)	12 (2.4)	84 (30.0)	16 (7.2)	51 (28.5)	49 (15.1)	67 (37.6)	33 (10.2)
Individual characteristics								
Age, years (median, IQR)	40 (33–48)		40 (33–48)	40 (33–48)	40 (34–48)	39 (32–47)	39 (32–46)	40 (34–49)
Minoritized sexual identity*	274 (54.5)		151 (53.9)	123 (55.2)	97 (54.2)	177 (54.6)	104 (58.4)	170 (52.3)
Minoritized gender identity*	77 (15.3)		38 (13.6)	39 (17.5)	23 (12.9)	54 (16.7)	22 (12.4)	55 (16.96)
Race		1 (0.2)						
White	191 (38.0)		108 (38.6)	83 (37.2)	76 (42.5)	115 (35.5)	66 (37.1)	125 (38.5)
Indigenous	280 (55.7)		160 (57.1)	120 (53.8)	99 (55.3)	181 (55.9)	104 (58.4)	176 (54.2)
Black or Woman of Colour^§^	31 (6.2)		11 (3.9)	20 (9.0)	ns	ns	ns	ns

For each model, the reference group includes both non-users of overdose prevention services and those who exclusively used the alternative service type. Specifically: (1) any overdose prevention services vs. no services; (2) community-based services vs. clinic-based or no services; and (3) clinic-based services vs. community-based or no services

^*^ In the last 6 months

^§^ Due to low sample size these categories were combined for analysis and protection of confidentiality

^†^ Excluding cannabis and alcohol

ns: Number suppressed due to low cell count to protect participant confidentiality

### Use of overdose prevention services over time (aim 1)

**Fig. 1:**
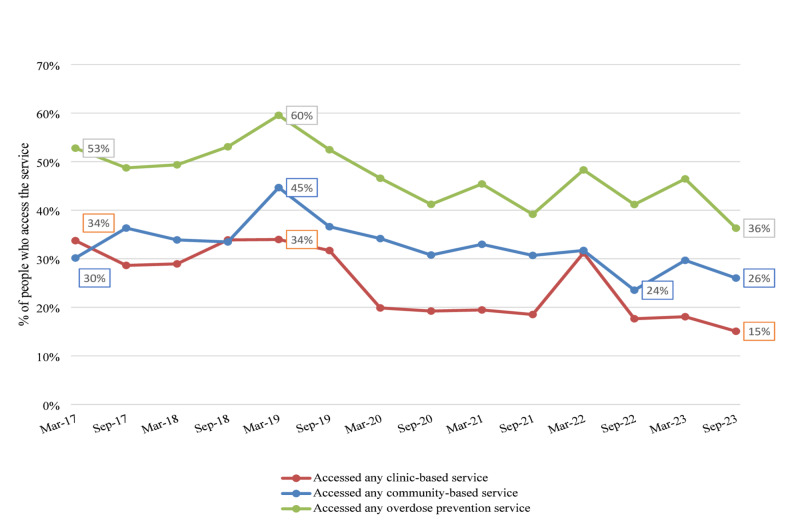
Biannual point prevalence of use of overdose prevention services among sex workers who use drugs Metro Vancouver, Canada (*N* = 503) AESHA, 2017–2024

Out of the study sample of 503 sex workers who use drugs, 82.1% (*N* = 413) reported using any overdose prevention service (either community or clinic-based) at least once during the 7-year study period. The proportion using community-based overdose services at least once was 70.2% (*N* = 353), and the proportion using clinic-based overdose prevention services at least once was 60.2% (*N* = 303). Use of overdose prevention services was highest between September 2018 and March 2019 and decreased during the initial phase of COVID-19 (Fig. 1). Time series regression analyses indicated sustained declines over the study period: any overdose prevention service use decreased by – 1.0% per six-month interval (95% CI – 1.7, – 0.4), community-based service use decreased by – 0.7% per interval (95% CI – 1.3, – 0.1), and clinic-based service use decreased by – 1.3% per interval (95% CI – 2.0, – 0.6). The observed rates of use did not return to pre-2019 levels during the remaining study period.

### Longitudinally characterize uptake of overdose prevention services (aim 2)

In bivariate GLMM analysis (Table 2), participants who experienced precarious housing (OR 2.01, 95%CI 1.60–2.51) or homelessness (OR 1.76, 95%CI 1.46–2.11) in the previous six months had higher odds of accessing any overdose prevention services. Among those experiencing homelessness, the odds of accessing clinic-based services only (OR 1.79, 95%CI 1.46–2.18) were slightly higher than the odds of accessing community-based services only (OR 1.67, 95%CI 1.37–2.03). Conversely, for participants experiencing precarious housing, the odds of accessing community-based services only (OR 2.28, 95%CI 1.65–3.15) exceeded the odds of accessing clinic-based services only (OR 1.68, 95%CI 1.30–2.18).

**Table 2:** Bivariate Generalized Linear Mixed Models (GLMM) analysis of access to any overdose prevention services, any community-based overdose prevention services, and any clinic-based overdose prevention services in the last 6 months among sex workers who use drugs in Metro Vancouver, Canada (*N* = 503), AESHA, 2017–2024

	Accessed any overdose prevention services (community or clinic)*	Accessed community-based overdose prevention services*	Accessed clinic-based overdose prevention services*
Characteristic	Unadjusted odds ratio (95%CI)	Unadjusted odds ratio (95%CI)	Unadjusted odds ratio (95%CI)
Structural determinants			
Precariously housed*	2.01 (1.60–2.51)	2.28 (1.65–3.15)	1.68 (1.30–2.18)
Homeless*	1.76 (1.46–2.11)	1.67 (1.37–2.03)	1.79 (1.46–2.18)
Primary place of solicitation*			
Indoor (formal or informal)	0.50 (0.36–0.70)	0.56 (0.38–0.81)	0.53 (0.36–0.78)
Independent	0.67 (0.54–0.83)	0.68 (0.55–0.85)	0.79 (0.65–0.96)
N/A–didn’t solicit in the last 6 months	0.52 (0.43–0.64)	0.61 (0.49–0.75)	0.52 (0.42–0.66)
Outdoor/public space (ref)			
Primary place of sex work service*			
Outdoor/public space	1.65 (1.35–2.01)	1.53 (1.23–1.91)	1.82 (1.46–2.26)
Indoor (formal or informal)	1.30 (1.08–1.55)	1.13 (0.92–1.38)	1.46 (1.21–1.76)
N/A–didn’t solicit in the last 6 months (ref)			
Used any drugs with client during a sex work transaction*	1.33 (1.14–1.55)	1.26 (1.06–1.49)	1.56 (1.32–1.84)
Worked with other sex workers for safety*			
Yes	1.10 (0.85–1.43)	1.05 (0.80–1.40)	1.35 (1.01–1.81)
N/A–didn’t actively work in the last 6 months	0.73 (0.61–0.86)	0.81 (0.67–0.97)	0.67 (0.55–0.81)
No (ref)			
Experienced violence from community members/businesses*	1.27 (0.98–1.65)	1.14 (0.85–1.52)	1.62 (1.25–2.11)
Experienced any physical/sexual violence from a client*	1.91 (1.37–2.66)	1.74 (1.27–2.40)	1.32 (0.93–1.88)
Experienced violence from negative police encounters*	1.45 (1.11–1.90)	1.34 (1.02–1.77)	1.53 (1.16–2.02)
Experienced any barriers to harm reduction due to police presence*	1.74 (1.41–2.15)	1.91 (1.54–2.36)	1.62 (1.31–2.00)
Enrollment in opioid agonist therapy*			
Yes	1.35 (1.12–1.61)	1.19 (0.99–1.44)	1.27 (1.04–1.55)
N/A–never used opioids	0.37 (0.26–0.55)	0.47 (0.32–0.69)	0.28 (0.18–0.46)
No (ref)			
Accessed drug/alcohol treatment*	2.00 (1.69–2.36)	1.79 (1.52–2.11)	1.62 (1.35–1.95)
Individual characteristics			
Age (per year older)	0.98 (0.97–0.99)	0.99 (0.98–1.00)	0.97 (0.95–0.98)
Minoritized sexual orientation*	1.24 (0.99–1.56)	1.12 (0.90–1.40)	1.34 (1.05–1.71)
Minoritized gender identity*	0.97 (0.74–1.26)	1.07 (0.82–1.41)	0.73 (0.51–1.05)
Racialization			
Indigenous ancestry	0.96 (0.76–1.22)	1.03 (0.81–1.30)	0.98 (0.76–1.28)
Black or Woman of Colour^§^	0.42 (0.25–0.70)	0.46 (0.28–0.77)	0.48 (0.25–0.93)
White (ref)			
Drug use patterns			
Overdosed*	1.87 (1.51–2.33)	1.68 (1.34–1.85)	1.63 (1.28–2.07)
Non–injection drug use†*	1.69 (1.43–2.01)	1.73 (1.43–2.11)	1.37 (1.13–1.66)
Injection drug use*	3.53 (2.91–4.28)	2.56 (2.14–3.06)	3.93 (3.13–4.93)
Needed injection assistance*	2.56 (2.06–3.19)	1.83 (1.49–2.25)	2.44 (2.00–2.98)

For each model, the reference group includes both non-users of overdose prevention services and those who exclusively used the alternative service type. Specifically: (1) any overdose prevention services vs. no services; (2) community-based services vs. clinic-based or no services; and (3) clinic-based services vs. community-based or no services

^*^ In the last 6 months

^§^ Due to low sample size these categories were combined for analysis and protection of confidentiality

^†^ Excluding cannabis and alcohol

Participants whose primary place of sex work was in outdoor/public settings had significantly higher odds of accessing any overdose prevention services (OR 1.65, 95%CI 1.35–2.01) compared to those who had not actively solicited in the past six months. They also had elevated odds of accessing community-based services (OR 1.53, 95%CI 1.23–1.91) and clinic-based services (OR 1.82, 95%CI 1.46–2.26). In contrast, those working in indoor settings had lower, though still increased, odds of accessing any overdose prevention services (OR 1.30, 95%CI 1.08–1.55), particularly clinic-based services (OR 1.46, 95%CI 1.21–1.76).

Experiencing occupational violence, drug use with clients, and accessing drug/alcohol treatment were also associated with increased odds of utilizing overdose prevention services. Participants who experienced physical or sexual violence from clients had higher odds (OR 1.91, 95%CI 1.37–2.66) of accessing any overdose prevention services, with significant associations for community-based services (OR 1.74, 95%CI 1.27–2.40). The effect was not statistically significant for clinic-based services (OR 1.32, 95%CI 0.93–1.88). Those who used drugs with clients had greater odds of accessing clinic-based services (OR 1.56, 95%CI 1.32–1.84) than community-based services (OR 1.26, 95%CI 1.06–1.49). Finally, participants who had accessed drug/alcohol treatment in the previous six months had twice the odds of accessing any overdose prevention services (OR 2.00, 95%CI 1.69–2.36).

### Use of sex worker-specific programs and use of overdose prevention services (aim 3)

In multivariable GLMM analysis using 2,610 observations from 484 participants (Table 3), the use of sex worker-specific programs was significantly and positively associated with access to overdose prevention services. In unadjusted analysis, using sex worker-specific programs was associated with higher odds of accessing any overdose prevention services (OR 2.28, 95%CI 1.93–2.70), community-based services only (vs. clinic-based or no services; OR 2.65, 95%CI 2.23–3.15), and clinic-based services only (vs. community-based or no services; OR 1.95, 95%CI 1.65–2.31). In multivariable GLMM analyses, use of sex worker-specific programs remained positively associated with access to overdose prevention services (AOR 2.18, 95%CI 1.82–2.62), with the strongest effects for community-based services only (AOR 2.66, 95%CI 2.21–3.20).

**Table 3:** Unadjusted and adjusted Generalized Linder Mixed-Model (GLMM) analysis for the association between use of sex work-specific programs and access to overdose prevention services (*N* = 484), AESHA, 2017–2024

	Outcomes					
	Accessed any overdose prevention services (community or clinic)*		Accessed community-based overdose prevention services*		Accessed clinic-based overdose prevention services*	
	Unadjusted	Adjusted	Unadjusted	Adjusted	Unadjusted	Adjusted
Exposure	Odds ratio (95%CI)	Odds ratio (95%CI)	Odds ratio (95%CI)	Odds ratio (95%CI)	Odds ratio (95%CI)	Odds ratio (95%CI)
Used sex worker-specific programs*	2.28 (1.93–2.70)	2.18 (1.82–2.62)	2.65 (2.23–3.15)	2.66 (2.21–3.20)	1.95 (1.65–2.31)	1.73 (1.41–2.13)

For each model, the reference group includes both non-users of overdose prevention services and those who exclusively used the alternative service type. Specifically: (1) any overdose prevention services vs. no services; (2) community-based services vs. clinic-based or no services; and (3) clinic-based services vs. community-based or no services

Adjusted for age, racialization, minoritized sexual identity, minoritized gender identity, experiences of homelessness, primary place of sex work service, experienced any physical/sexual violence from a sex work client, experienced violence from community members/businesses, drug use with a sex work client, and accessed drug/alcohol treatment, all in the last 6 months

^*^In the last 6 months

## Discussion

The escalating overdose crisis in BC and across North America underscores the urgent need for equitable access to evidence-based substance use and harm reduction services for people who use drugs [61]. In this seven-year study, 82% of sex workers who use drugs reported using overdose prevention services at least once, despite facing structural inequities such as unsafe working conditions, inadequate housing, police-related barriers to harm reduction efforts, and drug use with clients. While our findings underscore the strong reach of overdose prevention services among precariously housed and homeless sex workers, it is also important to recognize that employed or stably housed sex workers may face significant overdose risks that are less visible. As McDermid et al. [62] note, stigma and surveillance during the pandemic compounded overdose risks even in less publicly marginalized settings, underscoring that overdose risks are not confined to visibly precarious housing or street-based contexts. These risks, often shaped by discrimination in more hidden environments, may create unique barriers to harm reduction access and remain under-documented in the literature [63]. In adjusted analyses, participants who accessed sex worker-specific programs had 2.18 times higher odds of accessing any overdose prevention services (i.e., either community- or clinic-based services vs. no services), 2.66 times higher odds of accessing community-based services only (vs. clinic-based or no services), and 1.73 times higher odds of accessing clinic-based services only (vs. community-based or no services).

Trends in use of overdose prevention services indicate that during the seven-year study period use of services reached the highest point between September 2018 and March 2019 and then declined during the onset of COVID-19 where public health orders restricted access to many services, including harm reduction services [64, 65]. Recent work has documented the impact of COVID-19 responses in contributing to a heightened risk environment for sex workers who use drugs such as barriers to accessing essential health and safety supports due to closure of harm reduction services [66] or self-reliance to address overdose emergencies to avoid police interaction. These studies found that sex workers reported increases in occupationally related violence [67], and administering take-home naloxone (THN) to others for overdose reversal as well as facing increased odds of experiencing a non-fatal overdose themselves [62]. In this context, as well as in light of increased drug poisoning death rates in the post-pandemic period [61], it is concerning that trends in use of overdose prevention services did not return to pre-pandemic rates during the study period. These findings align with research among the broader population of people who use drugs in BC and internationally, where access to overdose prevention and harm reduction services declined during the COVID-19 pandemic and in many settings did not return to pre-pandemic levels [68–70]. This suggests that the service disruptions experienced by sex workers mirror wider trends in overdose risk environments. Looking forward, strengthening sustainable funding, scaling up hybrid and community-led service models, and implementing supportive policy reforms will be critical to ensure that overdose prevention services are resilient to future public health crises.

The patterns of use of overdose prevention services identified in this study complement and contrast previous quantitative and qualitative work in several ways. Previous work examining women’s experience accessing the clinic-based overdose prevention service InSite highlighted that accessing that space offered women a reprieve from violence which offered environmental and structural support to assert control over their drug consumption [71]. However, this reprieve was only afforded while women were inside the site. Once outside of the clinic-based environment, women returned to a street-based milieu that is characterized by high levels of violence [72]. Consistent with this finding, in this study sex workers who used clinic-based services were more likely to report violence from community members/businesses, although this association did not reach statistical significance when compared to individuals who either did not access any overdose prevention services or accessed community-based services. Clinic-based services are almost all entirely fixed, identifiable locations in mixed neighbourhoods with both commercial and residential properties. Accessing these spaces can heighten the visibility of sex workers and drug use. This visibility can exacerbate gendered street dynamics (i.e., the increased vulnerability and violence faced by women and gender-diverse people who use drugs in street settings) [73] as well as heightened police harassment in response to community complaints [74, 75].

When comparing study findings to prior research, complementary patterns were found for route of drug consumption. Compared with those accessing community-based services, a greater proportion of sex workers using clinic-based services reported injection drug use, needing injection assistance, as well as enrollment in opioid agonist therapy. These differences may be attributed to the classification of a supervised injection site (InSite) as a clinic-based service in this study, where nursing staff can provide guided instruction on safer injection practices [76]. While community-based services have demonstrated that peers can effectively assist with injection, often through manually operating the injection [77], most present-day community-based services are oriented to respond to inhalation rather than injection needs, which reflects broader trends in drug consumption changes locally [78].

Over the seven-year period, sex workers who reported homelessness or primarily working in outdoor/public settings had higher odds of accessing clinic-based overdose prevention services. This pattern may be explained by extended wraparound support and services offered through clinic-based settings. These include access to prepared meals, social service workers assisting with housing or other support services, and access to nursing or other medical supports for diverse health needs such as sexual health testing or other general health needs in addition to accessing addiction specialists for ongoing opioid agonist therapy treatment [12, 79, 80]. In contrast, while many community-based services may be more nimble and able to respond to changing drug use patterns these spaces tend to have a narrower focus of service provision [9]. Given that gendered experiences of homelessness are associated with extreme health inequities [26], access to more comprehensive services may be particularly important for women and gender-diverse sex workers. Moreover, gender dynamics within community-based overdose prevention services can influence service engagement, as many peer-staffed models are male-dominated outside of the limited number of women-only models, and may reproduce gender-based power dynamics [19, 46]. In the study’s local context, many low-barrier housing sites operate embedded community-based overdose prevention services, and qualitative research examining these sites notes that gender-based violence was a barrier to their use [47]. These findings emphasize the importance of housing-linked, gender-sensitive service models that address both safety and the intersection health and social needs of sex workers.

In this study, use of sex worker-specific programs was independently and positively associated with use of overdose prevention services across all three outcome models (i.e., use of any overdose prevention service; use of community-based *only*; and use of clinic-based *only*) over a seven-year period. The association across all three models remained robust after adjusting for structural factors including experiences of homelessness, primary place of sex work service, drug use with clients, occupational experiences of violence from community members/businesses, and access to drug/alcohol treatment. Programs dedicated to sex workers, especially those that meaningfully involved sex workers in their design, are better positioned to comprehensively respond to sex workers’ unique needs and their lived realities [37, 81]. These programs have long operated using a harm reduction approach, which aims to respond to human rights violations resulting from criminalization, stigma, and other institution repression [82], to reduce barriers to care and uphold sex workers’ right to non-discriminatory, affordable, culturally-specific services [83]. Further, through recognizing that sex work is work, these programs can empower sex workers to collectively challenge structural and interpersonal barriers to their labour and human rights [84]. Such approaches have been well documented to address inequities (e.g., HIV/STI burdens, other health concerns) [85, 86].

### Policy and program recommendations

Sex worker-specific programs play a crucial role in improving sex workers’ knowledge about health issues, including overdose risks, and inform them of services which can increase their confidence to seek care and recourse for rights violations [35, 41, 43, 87–89]. Community-based programs such as those offered by PACE Society and SWAN Vancouver are useful models to support scale-up [39, 40]. Our findings align with evidence that building awareness of resources and trust in peer-delivered education may lead to greater service utilization, as seen in literature documenting the effectiveness of peer-involved harm reduction initiatives for people who use drugs [18, 90, 91]. By creating environments of safety, trust, and inclusion, sex worker-specific programs offer tailored non-judgemental services tailored to sex worker’s needs and grounded in the right to self-determination [82]. This not only addresses individual health concerns, but also responds to broader structural barriers to accessing care such as stigma [84]. Building on previous evidence from diverse global settings, approaches that centre the needs of sex workers at the heart of programming models and facilitate safe, nonjudgmental support remain best practices for engaging with sex workers across multiple health and human rights concerns [37, 42, 92]. In practice, sex worker–inclusive and gender-responsive overdose prevention services across both clinical and community-based settings should incorporate elements such as peer navigators, confidential access points, drop-in hours, women-only or gender-specific spaces, trauma-informed screening and referrals, and co-located wraparound supports that address housing, mental health, and legal needs. More broadly, programmatic efforts to ensure that clinic and community-based overdose response programming are gender-sensitive and responsive to sex workers’ unique needs is also needed.

### Study limitations

While this study offers valuable insights into the relationships among structural factors shaping engagement with overdose prevention services among sex workers who use drugs, several limitations must be acknowledged. First, observed associations may be influenced by unmeasured confounding variables. Second, potential measurement biases may exist as the data relied on self-reports, which could introduce recall or social desirability biases. Because some sex worker-specific programs may also provide overdose prevention supports (e.g., naloxone distribution, overdose education), participants may have reported accessing both service types when these were co-located, which could have introduced misclassification and inflated observed associations. However, only a small number of sex worker-specific or led programs operated in Vancouver during the study period, nearly all located within the Downtown Eastside. These few services offered overdose prevention supports inconsistently over time, suggesting that such overlap likely occurred in a limited number of cases rather than representing the majority of program operations or timeframe. We were unable to disaggregate supervised drug consumption and drug checking services, as participant responses did not specify service type and stand-alone drug checking sites were not identified in our dataset. While this grouping reflects how services are often co-delivered in practice, future work assessing these service types separately is warranted, including examination of access to drug checking services as they continue to expand locally and internationally. British Columbia has been disproportionately impacted by the overdose crisis in Canada and has lengthier experience implementing drug-related interventions to respond to public health crises. Some clinic-based services in this study have been operational in the community for many years, and the integration of peers in many clinic-settings has been normalized. Many community-based services have been funded and amalgamated by regional health authorities. These contextual details may limit the generalization of the category definitions in other settings, where funding models may limit what services may be offered by community organizations [93, 94] and the use of different categories may be more appropriate. Finally, because each service type was modeled separately using binary GLMMs with a reference group that included both non-users and users of the alternate service type, direct comparisons between community- and clinic-based overdose prevention services should be interpreted with caution. Future research is recommended to examine which specific overdose prevention tools and strategies sex workers access in different overdose prevention service settings.

## Conclusion

This seven-year study found that despite facing significant structural barriers such as unsafe working conditions, homelessness, and police-related obstacles, the majority of sex workers in our cohort were actively involved in mitigating their risk of overdose-related harm through the use of overdose prevention services. Use of sex worker-specific programs were significantly linked to increased use of these services, especially community-based ones. Sex workers who experienced violence from a sex work client, reported police-related barriers to harm reduction, engaged in non-injection drug use were more likely to use community-based services. Whereas the use of clinic-based overdose prevention services was more likely among sex workers who reported violence from community members/businesses, injected drugs and accessed opioid agonist therapy. We recommend scaling up community-based, sex worker-specific and peer-delivered overdose prevention efforts, and expanding clinic-based models that are women-centered, trauma-informed, and sex worker-friendly, alongside broader structural reforms to decriminalize sex work and drug use. Continued promotion and integration of accessible, stigma-free clinic and community-based services are needed to meet the diverse needs of sex workers and other people who use drugs.

## Supplementary Information

Below is the link to the electronic supplementary material.

Supplementary Material 1.

## Data Availability

Data for this study are not publicly available for legal and ethical reasons, as this study involves sensitive data collected with a highly criminalized and stigmatized population of marginalized women. Under our current ethical approvals by the Providence Health Care—University of British Columbia (PHC-UBC) Institutional Research Ethics Board, de-identified data can be made available upon reasonable request and pending ethical approval. Please submit all requests to initiate the data access process to the corresponding author and PHC-UBC REB at ubc.all-reb@ubc.ca.

## Footnotes

1 The authors recognize the fluidity of gender and while cis and transfeminine gender identification was part of baseline enrollment criteria people can identify themselves in different ways at different times.

2 Given the low population of Black people in the Province of BC (less than 2%), the categories Black and Women of Colour were aggregated for analysis due to low sample size and the need to protect participant confidentiality.

3 Single room occupancy hotels (SROs) are often occupied as a last resort by marginalized community residents, characterized by significant health and safety violations and risks [57–59].

## References

[1] Tobias S, Grant CJ, Laing R, Arredondo J, Lysyshyn M, Buxton J, et al. Time-series analysis of fentanyl concentration in the unregulated opioid drug supply in a Canadian setting. Am J Epidemiol. 2022;191(2):241–7. 33977304 10.1093/aje/kwab129

[2] Kennedy MC, Dong H, Tobias S, Buxton JA, Lysyshyn M, Tupper KW, et al. Fentanyl concentration in drug checking samples and risk of overdose death in vancouver. Canada Am J Prev Med. 2024;66(1):10–7.37633426 10.1016/j.amepre.2023.08.016

[3] British Columbia Ministry of Health. Provincial health officer declares public health emergency. 2016 [cited 2023 Sep 3]. Available from: https://news.gov.bc.ca/releases/2016HLTH0026-000568

[4] United Nations Office on Drugs and Crime (UNODC). Reducing the adverse health and social consequences of drug abuse: a comprehensive approach. Vienna; 2009 [cited 2024 Oct 5]. Available from: //www.unodc.org/unodc/en/drug-prevention-and-treatment/publications/data/2009/november/reducing-the-adverse-health-and-social-consequences-of-drug-abuse_-a-comprehensive-approach.html

[5] Government of British Columbia. Overdose prevention. Province of British Columbia; 2022 [cited 2024 Jul 7]. Available from: https://www2.gov.bc.ca/gov/content/overdose/what-you-need-to-know/overdose-prevention

[6] British Columbia Ministry of Mental Health and Addictions. Escalating BC’s responses to the overdose emergency. Victoria BC; 2018 Feb. Available from: https://www2.gov.bc.ca/assets/gov/overdose-awareness/mmha_escalating_bcs_response_report_final_26feb.pdf

[7] Bardwell G, Fleming T, Collins AB, Boyd J, McNeil R. Addressing intersecting housing and overdose crises in Vancouver, Canada: opportunities and challenges from a tenant-led overdose response intervention in single room occupancy hotels. J Urban Health. 2019;96(1):12–20. 30073598 10.1007/s11524-018-0294-y PMC6391288

[8] Kerr T, Small W, Peeace W, Douglas D, Pierre A, Wood E. Harm reduction by a “user-run” organization: a case study of the Vancouver Area Network of Drug Users (VANDU). Int J Drug Policy. 2006;17(2):61–9.

[9] Wallace B, Pagan F, Pauly B. The implementation of overdose prevention sites as a novel and nimble response during an illegal drug overdose public health emergency. Int J Drug Policy. 2019;66:64–72. 30708237 10.1016/j.drugpo.2019.01.017

[10] Dogherty E, Patterson C, Gagnon M, Harrison S, Chase J, Boerstler J, et al. Implementation of a nurse-led overdose prevention site in a hospital setting: lessons learned from St. Paul’s Hospital, Vancouver, Canada. Harm Reduct J. 2022;19(1):13. 35120536 10.1186/s12954-022-00596-7 PMC8816684

[11] Clark AK, Wilder CM, Winstanley EL. A systematic review of community opioid overdose prevention and naloxone distribution programs. J Addict Med. 2014;8(3):153–63. 24874759 10.1097/ADM.0000000000000034

[12] Kennedy MC, Karamouzian M, Kerr T. Public health and public order outcomes associated with supervised drug consumption facilities: a systematic review. Curr HIV/AIDS Rep. 2017;14(5):161–83. 28875422 10.1007/s11904-017-0363-y

[13] Levengood TW, Yoon GH, Davoust MJ, Ogden SN, Marshall BDL, Cahill SR, et al. Supervised injection facilities as harm reduction: a systematic review. Am J Prev Med. 2021;61(5):738–49. 34218964 10.1016/j.amepre.2021.04.017 PMC8541900

[14] Potier C, Laprévote V, Dubois-Arber F, Cottencin O, Rolland B. Supervised injection services: what has been demonstrated? A systematic literature review. Drug Alcohol Depend. 2014;1(145):48–68. 10.1016/j.drugalcdep.2014.10.012 25456324

[15] Office of the Provincial Health Officer. Responding to British Columbia’s Public Health Emergency: Progress Update January to July 2020. 2020 [cited 2025 Sep 20]. Available from: https://www2.gov.bc.ca/assets/gov/health/about-bc-s-health-care-system/office-of-the-provincial-health-officer/overdose-response-progress-update-jan-july-2020.pdf

[16] British Columbia Centre for Disease Control. Dual Public Health Emergenices: Overdose in BC during COVID-10. 2021 [cited 2023 Sep 3]. Available from: http://www.bccdc.ca/resource-gallery/Documents/Statistics%20and%20Research/Statistics%20and%20Reports/Overdose/2021.04.16_Infographic_OD%20Dashboard.pdf

[17] Ray BR, Humphrey JL, Patel SV, Akiba CF, Bluthenthal RN, Tookes H, et al. Comparing harm reduction and overdose response services between community-based and public health department syringe service programmes using a national cross-sectional survey. Lancet Reg Health—Am. 2024 Jun 1 [cited 2024 Jul 7];34. Available from: https://www.thelancet.com/journals/lanam/article/PIIS2667-193X(24)00084-X/fulltext 10.1016/j.lana.2024.100757 PMC11091529 38745887

[18] Mercer F, Miler JA, Pauly B, Carver H, Hnízdilová K, Foster R, et al. Peer support and overdose prevention responses: a systematic ‘state-of-the-art’ review. Int J Environ Res Public Health. 2021;18(22):12073. 34831839 10.3390/ijerph182212073 PMC8621858

[19] Kennedy MC, Boyd J, Mayer S, Collins A, Kerr T, McNeil R. Peer worker involvement in low-threshold supervised consumption facilities in the context of an overdose epidemic in Vancouver. Canada Soc Sci Med. 2019;1(225):60–8. 10.1016/j.socscimed.2019.02.014 PMC6415769 30798157

[20] Goldenberg SM, Watt S, Braschel M, Hayashi K, Moreheart S, Shannon K. Police-related barriers to harm reduction linked to non-fatal overdose amongst sex workers who use drugs: Results of a community-based cohort in Metro Vancouver, Canada. Int J Drug Policy. 2020;1(76):102618. 10.1016/j.drugpo.2019.102618 PMC7673668 31838244

[21] Argento E, Shannon K, Fairbairn N, Moreheart S, Braschel M, Goldenberg SM. Increasing trends and incidence of nonfatal overdose in a cohort of women sex workers who use drugs in British Columbia: role of criminalization-related barriers to harm reduction. Drug Alcohol Depend. 2023;244:109789. 36753803 10.1016/j.drugalcdep.2023.109789 PMC10773461

[22] Emanuel E, Slater L, Croxford S, Edmundson C, Ibitoye A, Njoroge J, et al. Adverse health outcomes among people who inject drugs who engaged in recent sex work: findings from a national survey. Public Health. 2023;1(225):79–86. 10.1016/j.puhe.2023.09.024 37922590

[23] El-Bassel N, Norcini Pala A, Mukherjee TI, McCrimmon T, Mergenova G, Terlikbayeva A, et al. Association of violence against female sex workers who use drugs with nonfatal drug overdose in Kazakhstan. JAMA Netw Open. 2020;3(10):e2020802. 33044551 10.1001/jamanetworkopen.2020.20802 PMC7550967

[24] Harris MT, Goldenberg SM, Cui Z, Fairbairn N, Milloy MJS, Hayashi K, et al. Association of sex work and social-structural factors with non-fatal overdose among women who use drugs in Vancouver, Canada. Int J Drug Policy. 2023;1(112):103950. 10.1016/j.drugpo.2022.103950 PMC9974922 36640591

[25] Moreheart S, Shannon K, Krüsi A, McDermid J, Ettinger E, Braschel M, et al. Negative changes in illicit drug supply during COVID-19: associations with use of overdose prevention and health services among women sex workers who use drugs (2020–2021). Int J Drug Policy. 2023;1(121):104212. 10.1016/j.drugpo.2023.104212 PMC10798550 37797570

[26] Aldridge RW, Story A, Hwang SW, Nordentoft M, Luchenski SA, Hartwell G, et al. Morbidity and mortality in homeless individuals, prisoners, sex workers, and individuals with substance use disorders in high-income countries: a systematic review and meta-analysis. The Lancet. 2018;391(10117):241–50. 10.1016/S0140-6736(17)31869-X PMC5803132 29137869

[27] Shannon K, Strathdee SA, Goldenberg SM, Duff P, Mwangi P, Rusakova M, et al. Global epidemiology of HIV among female sex workers: influence of structural determinants. Lancet. 2015;385(9962):55–71. 25059947 10.1016/S0140-6736(14)60931-4 PMC4297548

[28] Strathdee SA, West BS, Reed E, Moazan B, Azim T, Dolan K. Substance use and HIV among female sex workers and female prisoners: risk environments and implications for prevention, treatment, and policies. JAIDS J Acquir Immune Defic Syndr. 2015;1(69):S110. 10.1097/QAI.0000000000000624 PMC4493865 25978477

[29] Argento E, Goldenberg SM, Shannon K. Preventing sexually transmitted and blood borne infections (STBBIs) among sex workers: a critical review of the evidence on determinants and interventions in high-income countries. BMC Infect Dis. 2019;5(19):212. 10.1186/s12879-019-3694-z PMC6399876 30832596

[30] Beckham SW, Glick JL, Schneider KE, Allen ST, Shipp L, White RH, et al. Latent classes of polysubstance use and associations with HIV risk and structural vulnerabilities among cisgender women who engage in street-based transactional sex in Baltimore City. Int J Environ Res Public Health. 2022;19(7):3783. 35409469 10.3390/ijerph19073783 PMC8997521

[31] Squires K. Sex workers in Canada face unequal access to healthcare: a systems thinking approach. J Prim Care Community Health. 2024;1(15):21501319241233172. 10.1177/21501319241233173 PMC10953055 38504526

[32] Iversen J, Long P, Lutnick A, Maher L. Patterns and epidemiology of illicit drug use among sex workers globally: a systematic review. In: Goldenberg SM, Morgan Thomas R, Forbes A, Baral S, editors. Sex work, health, and human rights: global inequities, challenges, and opportunities for action. Cham: Springer International Publishing; 2021. pp. 95–118. 10.1007/978-3-030-64171-9_6 36315784

[33] Abel G, Healy C. Sex worker-led provision of services in New Zealand: optimising health and safety in a decriminalised context. In: Goldenberg SM, Morgan Thomas R, Forbes A, Baral S, editors. Sex work, health, and human rights: global inequities, challenges, and opportunities for action. Cham: Springer International Publishing; 2021. pp. 175–87. 10.1007/978-3-030-64171-9_10 36315789

[34] Kerrigan D, Kennedy CE, Morgan-Thomas R, Reza-Paul S, Mwangi P, Win KT, et al. A community empowerment approach to the HIV response among sex workers: effectiveness, challenges, and considerations for implementation and scale-up. The Lancet. 2015;385(9963):172–85. 10.1016/S0140-6736(14)60973-9 PMC7394498 25059938

[35] Kim SR, Goldenberg SM, Duff P, Nguyen P, Gibson K, Shannon K. Uptake of a women-only, sex-work-specific drop-in center and links with sexual and reproductive health care for sex workers. Int J Gynaecol Obstet. 2015;128(3):201–5. 25627707 10.1016/j.ijgo.2014.09.026 PMC4329272

[36] Reza-Paul S, Steen R, Maiya R, Lorway R, Wi TE, Wheeler T, et al. Sex worker community-led interventions interrupt sexually transmitted infection/Human Immunodeficiency Virus transmission and improve Human Immunodeficiency Virus cascade outcomes: a program review from South India. Sex Transm Dis. 2019;46(8):556. 31295225 10.1097/OLQ.0000000000001020 PMC6629169

[37] World Health Organization, United Nations Population Fund, Joint United Nations Programme on HIV/AIDS, Global Network of Sex Work Projects, The World Bank. Implementing comprehensive HIV/STI programmes with sex workers: practical approaches from collaborative interventions. Geneva: World Health Organization; 2013 [cited 2022 Apr 27]. Available from: https://www.who.int/publications-detail-redirect/9789241506182

[38] Living in Community. Find a Service. 2024 [cited 2024 Nov 8]. Available from: https://livingincommunity.ca/find-a-service/

[39] PACE Society. What We Do. PACE Society. 2024 [cited 2024 Nov 8]. Available from: https://www.pace-society.org/what-we-do/

[40] SWAN Vancouver Society. What we do. SWAN Vancouver Society. n.d. [cited 2024 Nov 5]. Available from: https://swanvancouver.ca/what-we-do/

[41] Gibson K, Bowen R, Janssen P, Spittal P. Evaluation of the Mobile Access Project (MAP). Vancouver [British Columbia]; 2006. Available from: http://www.vancouveragreement.ca/wp-content/uploads/2006_EvaluationofMobileAccessProject.pdf

[42] Deering KN, Kerr T, Tyndall MW, Montaner JSG, Gibson K, Irons L, et al. A peer-led mobile outreach program and increased utilization of detoxification and residential drug treatment among female sex workers who use drugs in a Canadian setting. Drug Alcohol Depend. 2011;113(1):46–54. 20727683 10.1016/j.drugalcdep.2010.07.007

[43] Janssen PA, Gibson K, Bowen R, Spittal PM, Petersen KL. Peer support using a mobile access van promotes safety and harm reduction strategies among sex trade workers in Vancouver’s downtown eastside. J Urban Health. 2009;86(5):804–9. 19533367 10.1007/s11524-009-9376-1 PMC2729864

[44] Bardwell G, Austin T, Maher L, Boyd J. Hoots and harm reduction: a qualitative study identifying gaps in overdose prevention among women who smoke drugs. Harm Reduct J. 2021;18(1):1–10. 33678163 10.1186/s12954-021-00479-3 PMC7937364

[45] Boyd J, Collins AB, Mayer S, Maher L, Kerr T, McNeil R. Gendered violence and overdose prevention sites: a rapid ethnographic study during an overdose epidemic in Vancouver, Canada. Addiction. 2018;113(12):2261–70. 30211453 10.1111/add.14417 PMC6400212

[46] Boyd J, Lavalley J, Czechaczek S, Mayer S, Kerr T, Maher L, et al. “Bed bugs and beyond”: an ethnographic analysis of North America’s first women-only supervised drug consumption site. Int J Drug Policy. 2020;1(78):102733. 10.1016/j.drugpo.2020.102733 PMC7528035 32247720

[47] Collins AB, Boyd J, Hayashi K, Cooper HLF, Goldenberg SM, McNeil R. Women’s utilization of housing-based overdose prevention sites in Vancouver, Canada: an ethnographic study. Int J Drug Policy. 2020;1(76):102641. 10.1016/j.drugpo.2019.102641 PMC7114823 31887644

[48] Bardwell G, Kerr T, Boyd J, McNeil R. Characterizing peer roles in an overdose crisis: preferences for peer workers in overdose response programs in emergency shelters. Drug Alcohol Depend. 2018;1(190):6–8. 10.1016/j.drugalcdep.2018.05.023 PMC6091635 29960202

[49] Chang J, Shelly S, Busz M, Stoicescu C, Iryawan AR, Madybaeva D, et al. Peer driven or driven peers? A rapid review of peer involvement of people who use drugs in HIV and harm reduction services in low- and middle-income countries. Harm Reduct J. 2021;18(1):1–13. 33536033 10.1186/s12954-021-00461-z PMC7857348

[50] Shannon K, Bright V, Allinott S, Alexson D, Gibson K, Tyndall MW. Community-based HIV prevention research among substance-using women in survival sex work: the Maka Project Partnership. Harm Reduct J. 2007;4(1):1–6. 18067670 10.1186/1477-7517-4-20 PMC2248179

[51] Shannon K, Crago AL, Baral SD, Bekker LG, Kerrigan D, Decker MR, et al. The global response and unmet actions for HIV and sex workers. Lancet. 2018;392(10148):698–710. 30037733 10.1016/S0140-6736(18)31439-9 PMC6384122

[52] Goldenberg SM, Krüsi A, Zhang E, Chettiar J, Shannon K. Structural determinants of health among Im/migrants in the indoor sex industry: experiences of workers and managers/owners in metropolitan Vancouver. PLoS ONE. 2017;12(1):e0170642. 28141835 10.1371/journal.pone.0170642 PMC5283672

[53] Argento E, Goldenberg SM, Braschel M, Machat S, Strathdee SA, Shannon K. The impact of end-demand legislation on sex workers’ access to health and sex worker-led services: a community-based prospective cohort study in Canada. PLoS ONE. 2020;15(4):e0225783. 32251452 10.1371/journal.pone.0225783 PMC7135091

[54] Harris PA, Taylor R, Minor BL, Elliott V, Fernandez M, O’Neal L, et al. The REDCap consortium: building an international community of software platform partners. J Biomed Inform. 2019;95:103208. 31078660 10.1016/j.jbi.2019.103208 PMC7254481

[55] Harris PA, Taylor R, Thielke R, Payne J, Gonzalez N, Conde JG. Research electronic data capture (REDCap)—a metadata-driven methodology and workflow process for providing translational research informatics support. J Biomed Inform. 2009;42(2):377–81. 18929686 10.1016/j.jbi.2008.08.010 PMC2700030

[56] Güntner S. Precarious housing. In: Baikady R, Sajid SM, Przeperski J, Nadesan V, Islam MR, Gao J, editors. The Palgrave handbook of global social problems. Cham: Springer International Publishing; 2022. pp. 1–16. 10.1007/978-3-030-68127-2_174-1

[57] Shannon K, Ishida T, Lai C, Tyndall MW. The impact of unregulated single room occupancy hotels on the health status of illicit drug users in Vancouver. Int J Drug Policy. 2006;17(2):107–14.

[58] Knight KR, Lopez AM, Comfort M, Shumway M, Cohen J, Riley E. Single room occupancy (SRO) hotels as mental health risk environments among impoverished women: the intersection of policy, drug use, trauma, and urban space. Int J Drug Policy. 2014;25(3):556–61. 24411945 10.1016/j.drugpo.2013.10.011 PMC4014526

[59] General Manager of Arts and Culture and Community Services. SRO Update, 2023 Low-Income Housing Survey and Proposed SRA By-law Amendments. Vancouver [British Columbia]: City of Vancouver Council; 2023 May [cited 2024 Jun 9]. Available from: https://council.vancouver.ca/20230530/documents/r4.pdf

[60] Generalized Linear Mixed Models using Adaptive Gaussian Quadrature. [cited 2025 Mar 11]. Available from: https://drizopoulos.github.io/GLMMadaptive/

[61] BC Coroners Service. Unregulated Drug Deaths Summary. 2024 Feb [cited 2023 Aug 31]. Available from: https://app.powerbi.com/view?r=eyJrIjoiZTBlZDRmOGUtZGNjOC00NTVkLTliYTctYmQwNzMxMzNiZDE0IiwidCI6IjZmZGI1MjAwLTNkMGQtNGE4YS1iMDM2LWQzNjg1ZTM1OWFkYyJ9

[62] McDermid J, Pearson J, Braschel M, Moreheart S, Marck R, Shannon K, et al. Increases in housing rules and surveillance during COVID-19: impacts on overdose and overdose response in a community-based cohort of sex workers who use drugs in Vancouver, BC. Harm Reduct J. 2024;21(1):153. 39175071 10.1186/s12954-024-01030-w PMC11342539

[63] Ivsins A, MacKinnon L, Bowles JM, Slaunwhite A, Bardwell G. Overdose prevention and housing: a qualitative study examining drug use, overdose risk, and access to safer supply in permanent supportive housing in Vancouver. Canada J Urban Health. 2022;99(5):855–64. 36044156 10.1007/s11524-022-00679-7 PMC9430005

[64] Munro A, Booth H, Gray NM, Love J, Mohan ARM, Tang J, et al. Understanding the impacts of novel coronavirus outbreaks on people who use drugs: a systematic review to inform practice and drug policy responses to COVID-19. Int J Environ Res Public Health. 2021;18(16):8470. 34444219 10.3390/ijerph18168470 PMC8394531

[65] Vo AT, Patton T, Peacock A, Larney S, Borquez A. Illicit substance use and the COVID-19 pandemic in the United States: a scoping review and characterization of research evidence in unprecedented times. Int J Environ Res Public Health. 2022;19(14):8883. 35886734 10.3390/ijerph19148883 PMC9317093

[66] Bola R, Oviedo-Joekes E. At a crossroads: the intersecting public health emergencies of COVID-19 and the overdose crisis in BC. Br Columbia Med J. 2021 [cited 2024 Nov 5]. Available from: https://bcmj.org/blog/crossroads-intersecting-public-health-emergencies-covid-19-and-overdose-crisis-bc

[67] McBride B, Shannon K, Pearson J, Braschel M, Krüsi A, McDermid J, et al. Association between interrupted access to sex work community services during the COVID-19 pandemic and changes in sex workers’ occupational conditions: findings from a community-based cohort study in Vancouver, Canada. BMJ Open. 2023;13(1):e065956. 36604130 10.1136/bmjopen-2022-065956 PMC9826927

[68] Imtiaz S, Nafeh F, Russell C, Ali F, Elton-Marshall T, Rehm J. The impact of the novel coronavirus disease (COVID-19) pandemic on drug overdose-related deaths in the United States and Canada: a systematic review of observational studies and analysis of public health surveillance data. Subst Abuse Treat Prev Policy. 2021;16(1):87. 34844624 10.1186/s13011-021-00423-5 PMC8628272

[69] Cassie R, Hayashi K, DeBeck K, Milloy MJ, Cui Z, Strike C, et al. Difficulty accessing supervised consumption services during the COVID-19 pandemic among people who use drugs in Vancouver, Canada. Harm Reduct J. 2022;19(1):126. 36401299 10.1186/s12954-022-00712-7 PMC9675060

[70] Gubskaya E, Kennedy MC, Hayashi K, Cui Z, Milloy MJ, Kerr T. The impact of the COVID-19 pandemic on access to supervised consumption programs. Subst Abuse Treat Prev Policy. 2023;18(1):16. 36899417 10.1186/s13011-023-00521-6 PMC9999333

[71] Fairbairn N, Small W, Shannon K, Wood E, Kerr T. Seeking refuge from violence in street-based drug scenes: Women’s experiences in North America’s first supervised injection facility. Soc Sci Med. 2008;67(5):817–23. 18562065 10.1016/j.socscimed.2008.05.012

[72] McNeil R, Shannon K, Shaver L, Kerr T, Small W. Negotiating place and gendered violence in Canada’s largest open drug scene. Int J Drug Policy. 2014;25(3):608–15. 24332972 10.1016/j.drugpo.2013.11.006 PMC4031309

[73] Marshall BDL, Fairbairn N, Li K, Wood E, Kerr T. Physical violence among a prospective cohort of injection drug users: a gender-focused approach. Drug Alcohol Depend. 2008;97(3):237–46. 18487025 10.1016/j.drugalcdep.2008.03.028 PMC2570226

[74] Sherman SG, Tomko C, Silberzahn BE, White RH, Nestadt DF, Clouse E, et al. The role of local business employees and community members in the HIV risk environment of female sex workers in an urban setting: associations between negative interactions and inconsistent condom use. BMC Public Health. 2021;21(1):2265. 34895195 10.1186/s12889-021-12293-4 PMC8666055

[75] Ross BL. Sex and (evacuation from) the city: the moral and legal regulation of sex workers in Vancouver’s west end, 1975–1985. Sexualities. 2010;13(2):197–218.

[76] Kerr T, Mitra S, Kennedy MC, McNeil R. Supervised injection facilities in Canada: past, present, and future. Harm Reduct J. 2017;14(1):1–9. 28521829 10.1186/s12954-017-0154-1 PMC5437687

[77] McNeil R, Small W, Lampkin H, Shannon K, Kerr T. “People knew they could come here to get help”: an ethnographic study of assisted injection practices at a peer-run ‘unsanctioned’ supervised drug consumption room in a Canadian setting. AIDS Behav. 2014;18(3):473–85. 23797831 10.1007/s10461-013-0540-y PMC3815969

[78] George R, Steinberg A, Buxton JA. BC Harm Reduction Client Survey: Background and Significant Findings Throughout the Years. Vancouver [British Columbia]: BC Centre for Disease Control; 2021 [cited 2024 Oct 20]. Available from: http://www.bccdc.ca/Health-Professionals-Site/Documents/Harm-Reduction-Reports/Harm%20Reduction%20Client%20Survey%20Findings_May%202021.pdf

[79] Broadhead R, Kerr T, Grund JP. Safer injection facilities in North America: their place in public policy and health initiatives. J Drug Issues. 2002;1(32):329–55.

[80] Kerman N, Manoni-Millar S, Cormier L, Cahill T, Sylvestre J. “It’s not just injecting drugs”: supervised consumption sites and the social determinants of health. Drug Alcohol Depend. 2020;1(213):108078. 10.1016/j.drugalcdep.2020.108078 32485658

[81] Global Network of Sex Work Projects. Briefing Paper: The Meaningful Involvement of Sex Workers in the Development of Health Services Aimed At Them. 2017 [cited 2023 Jul 13]. Available from: https://www.nswp.org/sites/default/files/briefing_paper_meaningful_involvement_in_health_services_nswp_-_2017.pdf

[82] Santini T, Klein A, Stella, l’amie de Maimie, Butterfly Asian and Migrant Sex Worker Support Network. Sex Work and Harm Reduction Discourse. 2020. Available from: https://chezstella.org/wp-content/uploads/2020/09/Sex-Work-and-Harm-Reduction-Discourse.pdf

[83] Global Network of Sex Work Projects. NSWP Consensus Statement on Sex Work, Human Rights, and the Law. Global Network of Sex Work Projects. 2013 [cited 2024 Sep 22]. Available from: https://www.nswp.org/resource/nswp-publications/nswp-consensus-statement-sex-work-human-rights-and-the-law

[84] Gil CN, Ramaiah M, Mantsios A, Barrington C, Kerrigan D. Best practices and challenges to sex worker community empowerment and mobilisation strategies to promote health and human rights. In: Sex work, health, and human rights: global inequities, challenges, and opportunities for action. Springer; 2020. pp. 189–206 36315780

[85] Goldenberg SM, Thomas RM, Forbes A, Baral S, editors. Sex work, health, and human rights: global inequities, challenges, and opportunities for action. Vol. 15. 2022 [cited 2023 Aug 29]. Available from: https://www.emerald.com/insight/content/doi/10.1108/IJHRH-12-2022-214/full/html 36315662

[86] Johnson L, Potter LC, Beeching H, Bradbury M, Matos B, Sumner G, et al. Interventions to improve health and the determinants of health among sex workers in high-income countries: a systematic review. Lancet Public Health. 2023;8(2):e141–54. 36334613 10.1016/S2468-2667(22)00252-3 PMC10564624

[87] Abel G, Fitzgerald L, Brunton C. The impact of the prostitution reform act on the health and safety practices of sex workers. Christchurch: University of Otago; 2007.

[88] Benoit C, Belle-Isle L, Smith M, Phillips R, Shumka L, Atchison C, et al. Sex workers as peer health advocates: community empowerment and transformative learning through a Canadian pilot program. Int J Equity Health. 2017;16(1):160. 28854930 10.1186/s12939-017-0655-2 PMC5577770

[89] Busza J, Chiyaka T, Musemburi S, Fearon E, Davey C, Chabata S, et al. Enhancing national prevention and treatment services for sex workers in Zimbabwe: a process evaluation of the SAPPH-IRe trial. Health Policy Plan. 2019;34(5):337–45. 31157368 10.1093/heapol/czz037

[90] Marshall Z, Dechman MK, Minichiello A, Alcock L, Harris GE. Peering into the literature: a systematic review of the roles of people who inject drugs in harm reduction initiatives. Drug Alcohol Depend. 2015;151(1):1–14. 25891234 10.1016/j.drugalcdep.2015.03.002

[91] Parkes T, Matheson C, Carver H, Foster R, Budd J, Liddell D, et al. Assessing the feasibility, acceptability and accessibility of a peer-delivered intervention to reduce harm and improve the well-being of people who experience homelessness with problem substance use: the SHARPS study. Harm Reduct J. 2022;19(1):10. 35120539 10.1186/s12954-021-00582-5 PMC8815224

[92] Kerrigan DL, Fonner VA, Stromdahl S, Kennedy CE. Community empowerment among female sex workers is an effective HIV prevention intervention: a systematic review of the peer-reviewed evidence from low- and middle-income countries. AIDS Behav. 2013;17(6):1926–40. 23539185 10.1007/s10461-013-0458-4

[93] Kim HS, Aks SE. Take-home naloxone and the need for a publicly funded naloxone supply. J Addict Med. 2022;16(1):1–3. 33534277 10.1097/ADM.0000000000000821

[94] Stewart RE, Christian HP, Cardamone NC, Abrams C, Drob C, Mandell DS, et al. Mobile service delivery in response to the opioid epidemic in Philadelphia. Addict Sci Clin Pract. 2023;18(1):71. 38031174 10.1186/s13722-023-00427-5 PMC10687974

