# Supplementary material for: Use of clinic- and community-based overdose prevention services by sex workers who use drugs: findings from a community-based cohort in Vancouver, Canada (2017–2024)
Source: Harm Reduct J. 2026 Jan 27;23:39. doi: 10.1186/s12954-026-01398-x (PMC12918628; doi:10.1186/s12954-026-01398-x)
Supplement: Supplementary file 1 — Supplementary Material 1. [file 12954_2026_1398_MOESM1_ESM.docx]

**Supplementary Table 1. List of community-based and clinic-based overdose prevention services that were accessed by sex workers who use drugs in Metro Vancouver, Canada (N=503) AESHA, 2017-2024**

| Community Setting | Clinical Setting |
| --- | --- |
| Δ: service delivery taking place within a low-barrier location and typically operated by peer staff or community workers (non-medical/allied health professionals) | Δ: service delivery taking place within a location that is operated by a medical/allied-health professional and may or may not involve peers |
| 611 Powell / AESHA (community research office and drop-in centre staffed by experiential staff [people who use drugs and sex workers] and community workers) | Clinic |
| Alexander Street Community (low-barrier supportive housing) | Community physician |
| ANEKI (low-barrier women-only supportive housing) | Community police |
| Arco (low-barrier supportive housing) | Connections Clinic (outpatient addictions treatment centre) |
| ARYS / BCCSU (community research office and drop-in centre staffed by experiential staff [people who use drugs] and community workers) | Family Treatment |
| Biltmore (low-barrier supportive housing) | Hospital |
| Budzey (low-barrier supportive housing for women and women-led families) | InSite (supervised consumption site supporting injection drug use) |
| Building (low-barrier supportive housing) | Pharmacy |
| Carl Rooms (low-barrier supportive housing) | SafePoint (supervised consumption site supporting injection drug use) |
| Drop-in centre | Shoppers (pharmacy) |
| Flint (low-barrier supportive housing) | Street Nurse (outreach nursing program) |
| Front Room (Evelyne Saller Centre – drop-in centre) | Surrey Daytox (Creekside Withdrawal Management Centre - outpatient addictions treatment centre) |
| Hazelwood (low-barrier supportive housing) | Treatment Centre |
| Housing worker |  |
| Laura Secord (low-barrier women-only supportive housing) |  |
| Living Room (drop-in centre) |  |
| Lookout (emergency shelter and drop-in centre) |  |
| Maguerite Ford (low-barrier supportive housing) |  |
| MOPS (Molson Overdose Prevention Site) |  |
| New Fountain (emergency shelter and drop-in centre) |  |
| OPS (pop-up overdose prevention site tent) |  |
| Progressive Housing Society (low-barrier supportive housing) |  |
| Purpose (drop-in centre) |  |
| RainCity/Triage Shelter (emergency shelter and drop-in centre) |  |
| Red Door (low-barrier supportive housing) |  |
| Sereena’s (low-barrier women-only supportive housing) |  |
| Sister Space (women-only overdose prevention site) |  |
| Sorella’s (low-barrier supportive housing for women and women-led families) |  |
| Station Street (low-barrier supportive housing) |  |
| Tamura House (low-barrier supportive housing) |  |
| The Lux (low-barrier supportive housing) |  |
| The Maple (low-barrier supportive housing) |  |
| TORO Program (single room occupancy [SRO] tenant outreach program) |  |
| VANDU (drug user advocacy group office and drop-in centre) |  |
| Vivian (low-barrier women-only supportive housing) |  |
| Washington Window (needle exchange) |  |
